# Ebola outbreak in Uganda: urgent call for better prevention and surveillance

**DOI:** 10.1016/j.ebiom.2022.104366

**Published:** 2022-11-10

**Authors:** eBioMedicine

On Sept 20, health authorities in Uganda declared an outbreak of Ebola virus after a 24-year-old was confirmed positive for the relatively rare Sudan strain of the virus, termed Sudan Ebola virus, for which there are currently no licenced vaccines or treatments available.

According to the Ugandan Ministry of Health, a total of 60 confirmed cases, including 24 deaths and 24 recoveries, have been reported as of Oct 20. The current outbreak comes only 3 years since the last Ebola virus disease outbreak was announced in Uganda, back in 2019, and exactly a decade after the last outbreak owing to Sudan Ebola virus.

Ebola is a severe and often lethal haemorrhagic illness caused by the Ebola virus. Commonly believed to be of zoonotic origin, a typical Ebola virus disease outbreak starts with a single infected person, followed by transmission to other humans via direct contact or contact with infected bodily fluids or contaminated fomites. Ebolavirus belongs to the Filoviridae family of viruses, which also includes Cuevavirus and Marburgvirus. The genus *Ebolavirus* consists of six species, named *Sudan, Zaire, Bundibugyo, Tai Forest, Bombali*, and *Reston. Zaire Ebolavirus* (commonly known as the Ebola virus) and *Sudan Ebolavirus* are the dominant species that give rise to Ebola virus disease in humans.

One of the largest epidemics ever reported took place in west Africa between 2013 and 2016 and was due to the Ebola virus. It resulted in over 28 600 suspected cases and more than 11 300 confirmed deaths. This epidemic was the first Ebola virus disease outbreak in which the ERVEBO vaccine, developed by Merck (Kenilworth, NJ, USA), was widely distributed among the affected communities, resulting in a prevention rate of over 80%. A clinical trial published in *The New England Journal of Medicine* that was conducted during the same outbreak found that monoclonal antibody treatments mAb114 (Ansuvimab, sold under the brand name Ebanga) and REGN-EB3 (Inmazeb) were effective at reducing mortality from Ebola virus disease. Both are now included in the first guidelines recommended by WHO for Ebola virus disease therapeutics. However, similar therapies and vaccines to prevent the spread of the current Sudan Ebola virus outbreak are still in various stages of clinical development and no date has been issued for availability. This delay is in part due to the rarity of Sudan Ebola virus outbreak and researchers not being able to widely test some of these candidates.

Several vaccine candidates have been evaluated against Sudan Ebola virus in phase 1 human trials, including virus-like particles, recombinant modified vaccinia virus, DNA vaccines, human and chimpanzee adenoviruses, and viral vectors (eg, vesicular stomatitis virus). As of October, three vaccine candidates have passed safety testing in humans, and six more are in the pipeline. But the efficacy of these candidates has yet to be tested in a large-scale study. The one furthest in development is a replication-defective recombinant chimpanzee adenovirus type 3-vectored ebolavirus vaccine (cAd3-EBO), encoding glycoprotein from the Ebola virus and Sudan Ebola virus. A phase 1 trial published on Nov 26, 2014, in *The New England Journal of Medicine* assessed its safety and immunogenicity and found that the cAd3-EBO vaccine was well tolerated with no serious safety concerns, and vaccine-induced immune responses were similar to findings from previous challenge studies from non-human primates, and sustained responses were evident for up to 11 months. Several thousand doses of this vaccine, which is licensed to the Sabin Vaccine Institute (Washington DC, USA) are anticipated for imminent potential deployment in Uganda, pending local regulatory approval for an efficacy trial during the current outbreak.

Another strong contender that is being geared for efficacy testing during the current outbreak in Uganda is a two dose heterologous booster vaccine consisting of a prime dose of adenovirus type 26 vector encoding Ebola glycoprotein (Ad26.ZEBOV), followed by a secondary dose of modified vaccinia Ankara vector, encoding glycoproteins from the Ebola virus, Sudan Ebola virus, Marburg virus, and Tai Forest virus nucleoprotein (MVA-BN-Filo), with a 56-day gap. The vaccine was developed as part of the EBOVAC project, which is a research and pharmaceutical partnership aimed at forming a strategic alliance to accelerate the development of Ebola vaccines via clinical trials or following regulatory approval. Earlier phase 1 studies were conducted to assess the safety and tolerability of this vaccine regimen and potential long-term durability of the vaccine-induced immune responses. The latest results on the safety and long-term immunogenicity of this vaccine regimen were shown in a trial published in the January 2022 issue of *The Lancet Infectious Diseases*. The trial was conducted in Sierra Leone, a country previously affected by Ebola, extending their data collected from several other countries, including Uganda, Tanzania, Kenya, and the UK. This vaccine regimen received marketing authorisation for prophylactic use under exceptional circumstances in the EU as the trial results showed that Ebola virus glycoprotein-specific antibody binding at 21 days after the second dose were similar with levels observed after 21 days with rVSV-ZEBOV-GP vaccine. The latter is the only vaccine with demonstrable efficacy data in the world that has marketing authorisation in the EU, USA, and several African countries.

While these vaccine trials are being set up, the Ugandan Government is mobilising several different surveillance and containment measures to curtail the virus from spreading to highly populated regions that are based on lessons learnt from previous Ebola virus disease outbreaks. On Oct 15, Uganda's President Yoweri Museveni introduced a 3 week lockdown in two affected districts of Uganda, along with a curfew to restrict movement of individuals, as a control measure to halt the transmission of Sudan Ebola virus to other regions and neighbouring countries. The country's Ministry of Health has ramped up training for health-care workers to handle Sudan Ebola virus case management, from contact tracing to safe burials of the dead and decontamination of affected dwellings, and training local volunteers to educate rural communities about the disease and its management. Contact tracing is being widely deployed to identify individuals who have been in close contact with affected individuals, who are then monitored for up to 21 days. Bordering countries, among others (including the USA), are on high alert and are implementing surveillance measures at border controls to curb international spread of the virus.

A lesson learnt from the COVID-19 pandemic is also being put into practice, whereby a mobile laboratory has been set up in Mubende, where the outbreak first began, to enable on site, same day diagnosis. Several diagnostic methods, including RT-PCR, can be used to diagnose Sudan Ebola virus. However, the early signs of the disease mimic other infectious diseases, such as malaria, typhoid fever, and meningitis, which adds to the challenges of running diagnostics in remote low-resource setting. A study published in *The Lancet Infectious Diseases* by Mukadi-Bamuleka and colleagues developed and evaluated the performance of three rapid diagnostic tests (RDT) for detecting viral antigens as a means to overcome some of these diagnostic challenges in a remote setting. Despite robust performance, all three RDTs, including the OraQuick, Coris EBOLA Ag K-SeT and QuickNavi-Ebola, failed to reach WHO's recommended threshold for sensitivity and specificity and did not show similar performance to the Cepheid GeneXpert Ebola assay reference test. These disappointing findings highlighted the need for the development of robust tests that are affordable, can provide a rapid diagnosis, and can be used with minimal training in a remote setting.

In a study by Ravichandran and Khurana published in *eBioMedicine*, the authors developed an ELISA-based serodiagnostic assay, referred to as EBOV-Detect, that can differentiate Ebola virus disease-induced antibodies from those that have been raised in response several candidate vaccines (namely rVSV-ZEBOV and ChAd/MVA-EBOV) with 95% specificity and 96% sensitivity. The authors hope that this assay, and other similar diagnostic tools, will be implemented in later stages of the outbreak to facilitate epidemiological and surveillance efforts.

In the absence of effective treatments and vaccines, the Ugandan Government, backed by international health authorities, can end the Sudan Ebola virus outbreak by applying all the lessons learnt through past experiences, such as surveillance strategies and timely response management tailored to the affected communities on the ground. At *eBioMedicine*, we encourage researchers working on all aspects of Ebola virus disease diagnostic, surveillance, and epidemiological and disease mechanisms to submit robust and early evidence to form studies to help fight the Ebola virus disease outbreak.

